# Acetyltransferase p300 inhibitor reverses hypertension‐induced cardiac fibrosis

**DOI:** 10.1111/jcmm.14162

**Published:** 2019-02-01

**Authors:** Rahul Rai, Tianjiao Sun, Veronica Ramirez, Elizabeth Lux, Mesut Eren, Douglas E. Vaughan, Asish K. Ghosh

**Affiliations:** ^1^ Feinberg School of Medicine Feinberg Cardiovascular and Renal Research Institute, Northwestern University Chicago IL

**Keywords:** acetyltransferase p300, cardiac fibrosis, cardiac hypertrophy, collagens, epigenetics, hypertension, small molecule inhibitors of p300

## Abstract

Epigenetic dysregulation plays a crucial role in cardiovascular diseases. Previously, we reported that acetyltransferase p300 (ATp300) inhibitor L002 prevents hypertension‐induced cardiac hypertrophy and fibrosis in a murine model. In this short communication, we show that treatment of hypertensive mice with ATp300‐specific small molecule inhibitor L002 or C646 reverses hypertension‐induced left ventricular hypertrophy, cardiac fibrosis and diastolic dysfunction, without reducing elevated blood pressures. Biochemically, treatment with L002 and C646 also reverse hypertension‐induced histone acetylation and myofibroblast differentiation in murine ventricles. Our results confirm and extend the role of ATp300, a major epigenetic regulator, in the pathobiology of cardiac hypertrophy and fibrosis. Most importantly, we identify the efficacies of ATp300 inhibitors C646 and L002 in reversing hypertension‐induced cardiac hypertrophy and fibrosis, and discover new anti‐hypertrophic and anti‐fibrotic candidates.

## 1 INTRODUCTION

Cardiovascular diseases are the number one cause of all disease‐related deaths worldwide. Sustained high blood pressure causes cardiac hypertrophy and myocardial fibrosis, leading to loss of myocardial elasticity and heart failure.^1^ As there is no available therapy for regression of cardiac fibrosis, it is imperative to identify a potential druggable target and its modulators for an efficient therapy. Epigenetics plays a key role in cellular physiology and pathology by regulating gene expression via acetylation, deacetylation, methylation, demethylation and miRNA‐mediated translational regulation.^2^ Acetyltransferase p300 (ATp300), a major epigenetic regulator, controls numerous human cellular activities during development and its dysregulation is associated with numerous diseases including cancers, Rubinstein Taybi syndrome and long‐term memory impairment.^3^, ^4^, ^5^, ^6^, ^7^ We were the first to demonstrate that extracellular matrix protein Type I collagen, major contributor in organ fibrogenesis, is epigenetically regulated by ATp300, a non‐DNA binding protein with intrinsic acetyltransferase activity and different functional domains [reviewed in^8,9^]. This novel study documented that elevated levels of ATp300 lead to an increased synthesis of collagen by TGF‐β‐activated fibroblasts/myofibroblasts. Most importantly, TGF‐β, the most potent profibrogenic cytokine, fails to stimulate Type I collagen synthesis in ATp300 depleted fibroblasts even in the presence of ATp300‐related CBP. These results demonstrate the essential role of ATp300 in profibrogenic signal‐induced collagen synthesis [reviewed in 8, 9]. Following this novel report, numerous studies confirmed and established the pivotal role of ATp300 in fibrogenic signalling and pathogenesis of organ fibrosis [reviewed in 2, 9]. Additionally, it is apparent that collagen promoter is not addicted for CBP in p300 depleted fibroblasts as has been shown for addiction of Myc gene promoter for CBP in p300 deficient cancer cells and for p300 in CBP deficient cancer cells.^10^ More recently, we showed that L002, a potent inhibitor of p300, prevents hypertension‐induced cardiac hypertrophy and fibrogenesis.^11^ In this study, we also demonstrated the effectiveness of C646 and L002 in blocking TGF‐β‐induced profibrogenic signalling in vitro.^11^ However, it is not known whether pharmacological inhibition of ATp300 halts cardiac fibrogenesis or reverses established fibrosis more relevant to drug development and effective therapy. Here, we tested the efficacies of two ATp300 inhibitors, L002 and C646, in reversing hypertension‐induced established cardiac fibrogenesis in vivo.

## 2 MATERIALS AND METHODS

### 2.1 Generation of hypertensive mice and post‐treatment with ATp300 inhibitors C646 and L002

**Figure 1:**
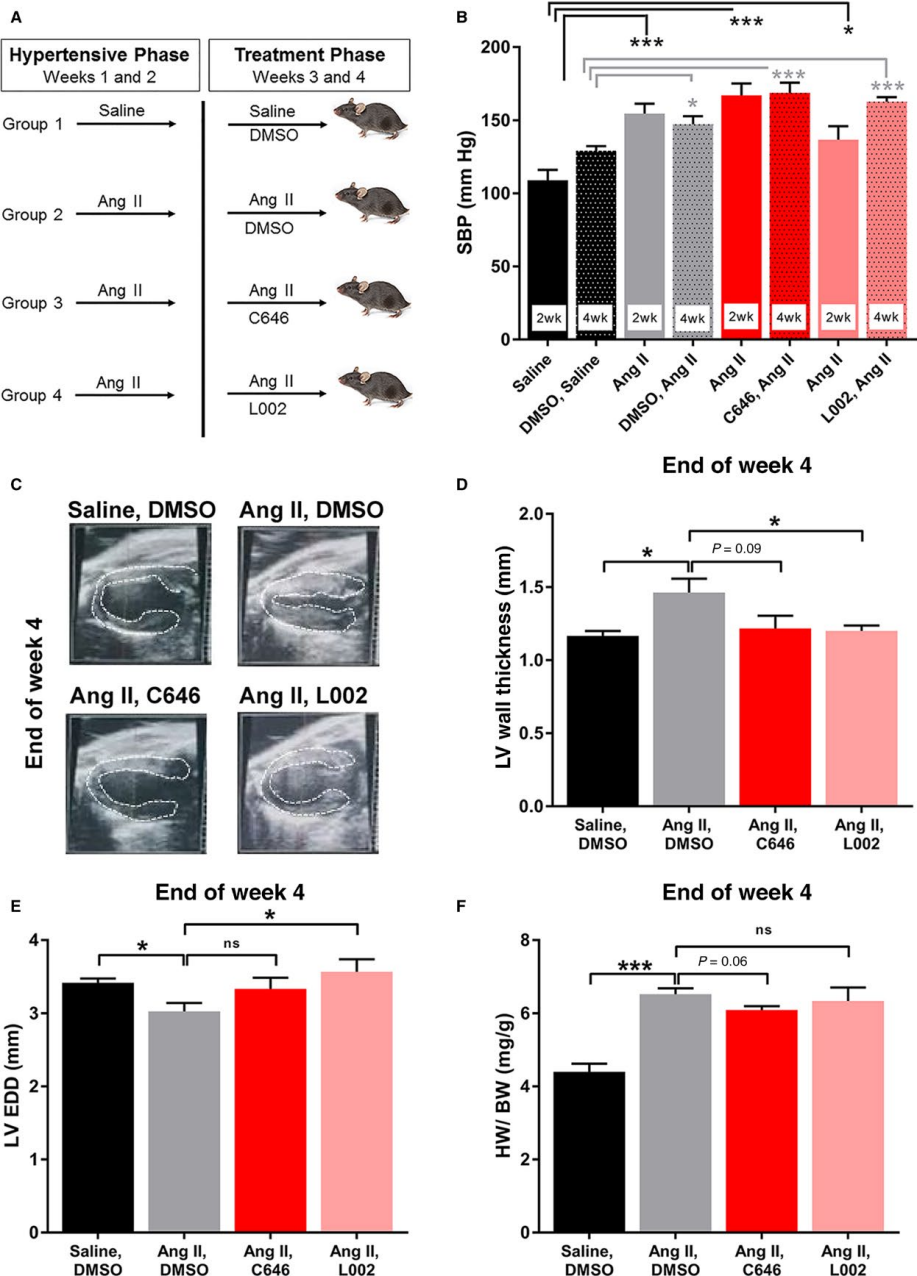
Post‐treatment of hypertensive mice with ATp300 inhibitors reverse hypertension‐induced ventricular wall thickness. Hypertension was induced by minipump‐mediated infusion of Angiotensin II in wild‐type mice. Minipump‐mediated saline infused mice were used as normotensive control. Angiotensin II‐induced hypertensive mice were treated with either DMSO (Group 2; n = 6); or C646 (Group 3, n = 6); or L002 (Group 4; n = 6) for 2‐wk on every 3rd day by intraperitoneal injection after 2 wk of saline or Angiotensin II minipump implantation. Saline‐infused mice were treated with DMSO (control) (Group 1; n = 6) (A). Blood pressures were recorded on weeks 2 and 4 (B) using tail‐cuff method. Echocardiogram was performed on week 4. Echocardiography showing images of the left ventricular wall thickness (LVWT) (C) and quantification data (D). The quantitative data of left ventricular end diastolic diameter (LVEDD) in four groups are shown in E. Heart weight to body weight are presented in F. ns, not significant; *P < 0.05; ***P < 0.005

Eight weeks old wild‐type C57BL/6 male mice were purchased from Jackson laboratory, Bar Harbor, ME and were maintained. All mouse protocols were approved by the Northwestern University Animal Care and Use Committee. To generate hypertensive mice, osmotic minipumps (Alzet‐200; Durect Corp., CA) were implanted on the back of mice (n = 6) to infuse Angiotensin II (Ang II, Bachem, Torrance, CA, USA; 1500 ng/kg/min for 4 weeks). Saline‐infused mice were used for normotensive control group [for details see 11]. After 2 weeks of Angiotensin II or saline infusion, batches of mice received L002 or C646 (Sigma Chemicals, USA) (~20 μg/gm body weight) by intraperitoneal injection on every 3rd day for another two weeks. The small molecule inhibitor, L002 is a distinct ATp300 inhibitor with two ring moieties where quinone imine and methyoxyphenyl groups are connected by sulfonyl group. C646 is a pyrazolone‐containing small molecule inhibitor of ATp300. Both L002 and C646 fit well in the acetyl‐CoA pocket of ATp300 catalytic domain and thus L002 and C646 are competitive inhibitors. These small molecules are cell permeable and reversible inhibitors. Control group received equal volume of vehicle (DMSO) (Figure 1A). The mice were sacrificed and hearts were collected after 4 weeks. A part of the heart was fixed in formalin and used for histochemical analysis. Other half of the heart was snap frozen and stored at −80°C for biochemical analysis.

### 2.2 Blood pressure measurement and echocardiography

The blood pressures on week 2 and 4 were recorded by tail‐cuff method. The body weight of mice in all four groups was recorded. After 4 weeks, trans‐thoracic two‐dimensional M‐mode echocardiography was performed as described.^11^ M‐mode tracings were used to measure the effects of ATp300 inhibitors L002 and C646 on murine cardiac structure and function. The left ventricular wall thickness (LVWT), left ventricular end diastolic diameter (LVEDD) and ejection fractions were determined. The mean value of at least 3‐5 cardiac cycles were used to determine the measurements for each animal in hypertensive groups treated with or without ATp300 inhibitor L002 or C646. Post‐mortem heart weight in all mice was recorded to analyse the ratio of heart weight to body weight.

### 2.3 Masson's trichrome staining for collagen

Paraffin‐embedded heart tissues from all four groups were subjected to microtome sectioning and processed for Masson's trichrome staining to detect the levels of collagen deposition in hearts.^11^ Photographs were taken with an Olympus DP71 camera. The extent of interstitial and perivascular fibrosis in heart sections from saline +DMSO controls (n = 6), Ang II +DMSO (n = 6), Ang II +C646 (n = 5), and Ang II +L002 mice (n = 6) were quantified by free‐hand drawing and measured the area of interstitial fibrotic areas relative to total cardiac cross section areas (interstitial fibrosis in %) or perivascular fibrotic areas relative to blood vessels areas (relative perivascular fibrosis) and analysed with ImageJ‐1.52a software (NIH). The effects of ATp300 inhibitors C646 and L002 on Ang II‐induced cardiomyocyte hypertrophy were determined by free‐hand tracing the areas of cardiomyocytes in the non‐fibrotic areas (photographs taken in 60X magnification) and measurement using ImageJ‐1.52a software (NIH) in all four groups. Approximately the areas of 80‐100 cardiomyocytes were measured per mouse (n = 6/group) and used for statistical analysis.

### 2.4 Preparation of myocardial tissue lysates and Western blot analysis

Ventricular tissue lysates were prepared using RIPA lysis buffer (Thermo Fisher Scientific, Waltham, MA) with protease and phosphatase inhibitors (Sigma, St Louis, MO). Ventricular tissues were homogenized in lysis buffer using Pestle Motor Mixer (Argos Technologies) and pipetted several times on ice. Equal amount of protein from hearts (n = 6) in each group was pooled and subjected to gradient gel electrophoresis and Western blot analysis using antibodies against α‐SMA (Sigma, St Louis, MO), Ac‐H3K9 (Cell Signaling, Danvers, MA) and α‐tubulin (GenScript, Piscataway, NJ).

### 2.5 Cell culture, treatment and Western blot

Human cardiac fibroblasts were cultured in 10% FBS containing DMEM media. Cultures of cardiac fibroblasts in 12‐well clusters were treated in triplicate with ATp300 inhibitor C646 (10 µM) (Sigma Chemicals, MO) or vehicle in the presence or absence of TGF‐β2 (10 ng/ml) for 48 hours. Experiments were repeated two times. Cell lysates were prepared using RIPA lysis buffer (ThermoFisher Scientific) with protease and phosphatase inhibitors (Sigma, MO) and pooled from three wells. Equal amount of proteins was subjected to Western blot using p53 (Santa Cruz Biotech.) and Actin (Abcam) antibodies.

### 2.6 Statistical analysis

Data are presented as Mean ± SEM. The significance of differences between controls and experimental groups was estimated by *t* test or two‐way ANOVA and a value of *P* < 0.05 by Student *t *test was considered statistically significant. Statistical analyses were performed with GraphPad Prism (GraphPad Software Inc, San Diego, CA).

## 3 RESULTS AND DISCUSSION

Pathological matrix remodelling and loss of contractility in the heart is the major cause of cardiovascular disease‐related deaths. There is no effective therapy to halt or reverse established cardiac fibrogenesis. Here, we examined the effectiveness of two small molecule inhibitors of ATp300, L002 and C646 in reversing hypertension‐induced cardiac hypertrophy and fibrosis by treatment of mice after inducing hypertension for two weeks. The present approach is significant as reversal of cardiac hypertrophy and fibrosis is more relevant for developing newer epigenetic therapies targeting ATp300. We began our assessment by measuring blood pressure after 2 weeks of Ang II infusion. As expected, Ang II infusion induced a hypertensive response in all the three groups (Figure 1A,B). After induction of hypertension for 2 weeks, mice in groups 3 and 4 were treated with C646 and L002 by intraperitoneal injection respectively. Interestingly, co‐treatment for last 2 weeks with C646 or L002 had no effect on blood pressure as high SBP were measured at the end of week 4 (Figure 1B).

Next, we determined the effects of ATp300 inhibitors post‐treatment on hypertension‐induced cardiac structure and function. Echocardiographic analysis revealed that Ang II significantly increased the thickness of left ventricular wall. Importantly, last 2 week co‐treatment of ATp300 inhibitors C646 or L002 significantly reduced or normalized LVWT despite continuous infusion of Ang II (Figure 1C,D). Cardiomyocyte area measurement data revealed that the cardiomyocytes areas are significantly increased in response to Ang II compared to saline infused controls as expected. Most importantly, C646 and L002 post‐treatments reduce the Ang II‐induced cardiomyocyte size (Figure S1A,B). Echocardiographic analysis further reveal that while C646 post‐treatment partly reverses the hypertension‐induced decreased LVEDD, L002 post‐treatment significantly reverses decreased LVEDD (Figure 1E). Although diastolic function was not specifically assessed by measuring mitral valve E/A ratio, reduction in LVEDD with Ang II treatment demonstrates diastolic dysfunction secondary to ventricular stiffening. Taken together, these data suggest that inhibition of ATp300 by either C646 or L002 after 2 weeks of Ang II infusion reverses hypertension‐induced pathological cardiac hypertrophy and cardiac remodelling, and the beneficial effects of ATp300‐specific inhibitors are independent of increased blood pressures. Based on the ejection fraction data, systolic dysfunction was not evident in this model and was unaltered by post‐treatment with ATp300 inhibitors L002 and C646 (Figure S2). Post‐mortem analysis revealed that Ang II‐induced hypertension increased heart weight to body weight as expected. However, co‐treatment of mice with ATp300 inhibitors C646 or L002 for last 2 weeks had no significant effect on cardiac weight (Figure 1F).

**Figure 2:**
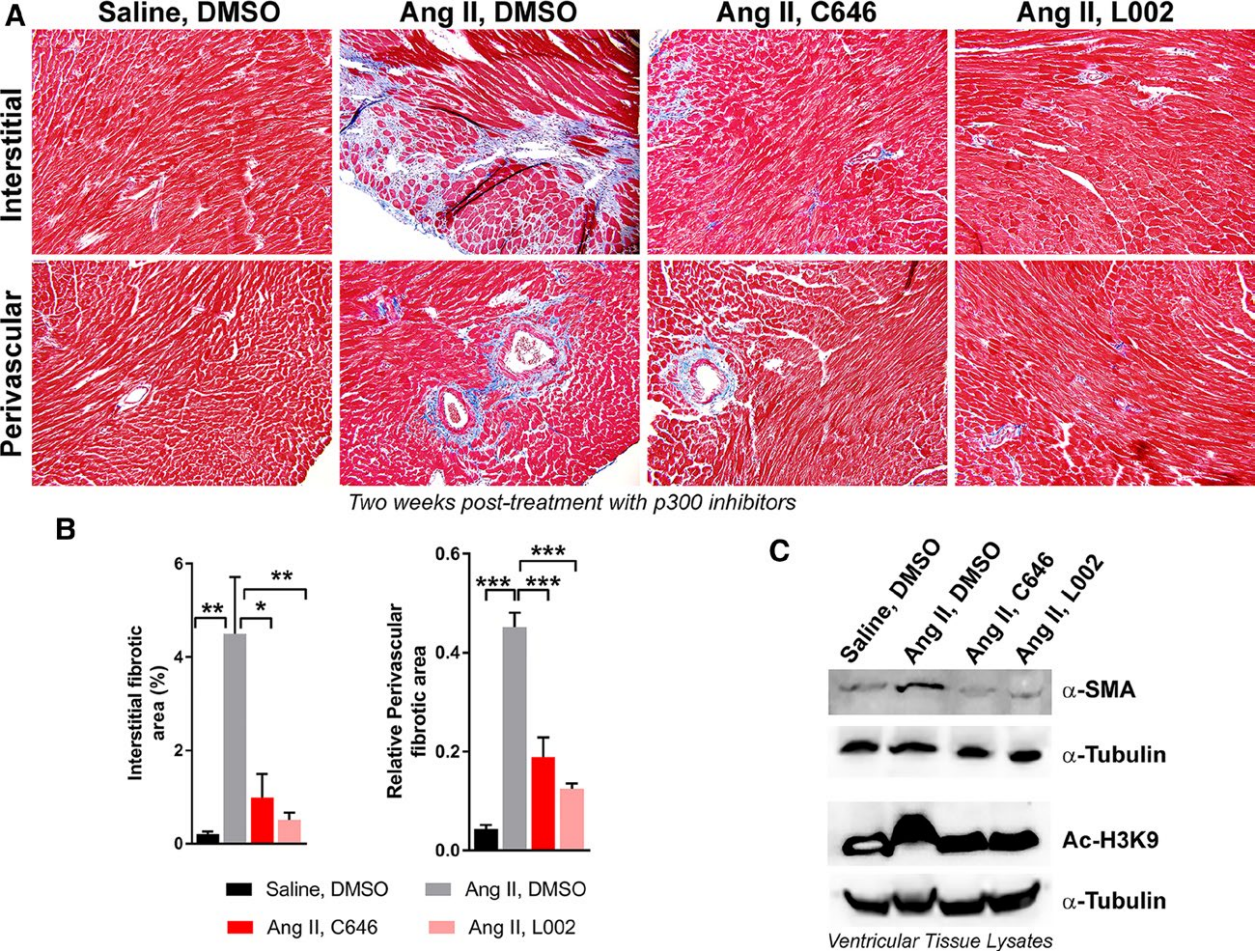
ATp300 inhibitors effectively reverse Angiotensin II‐mediated hypertension‐induced myofibroblast differentiation and cardiac fibrosis. Batches of Angiotensin II‐infused mice were post‐treated with either DMSO (Group 2; n = 6); or C646 (Group 3, n = 5); or L002 (Group 4; n = 6). Saline‐infused mice were treated with DMSO (control) (Group 1; n = 6) as described in Figure 1A legend. After 4 wk, the hearts were collected and processed for immunohistochemical analysis. Masson's trichrome staining for perivascular and interstitial collagen deposition (A); Quantification of interstitial and perivascular fibrotic areas using ImageJ 1.52a software as described under methods (B). *P < 0.05; **P < 0.01; ***P < 0.005. Effect of C646 and L002 on hypertension‐induced α‐SMA expression and the levels of Ac‐H3K9 in myocardial tissue lysates pooled from six hearts in each group (n = 6) and subjected to Western blot analysis using antibodies against α‐SMA, Ac‐H3K9 and α‐Tubulin (C)

Next, we investigated the effects of C646 and L002 post‐treatment on hypertension‐induced cardiac matrix remodelling. Analysis of Masson's Trichrome staining of ventricular sections from all four groups revealed that hypertension significantly increased deposition of collagen in the perivascular and interstitial areas of myocardial tissues. However, post‐treatment of hypertensive mice with C646 or L002, significantly reduces the levels of perivascular and interstitial collagen in the myocardium compared to non‐treated hypertensive mice (Figure 2A,B). These results explicitly establish the effectiveness of ATp300 inhibitors in reversing pathological cardiac matrix remodelling close to physiological levels in hypertensive mice. Our biochemical analysis (Immunoblot) of ventricular tissue extracts from six hearts in each group revealed that hypertension induces myofibroblast differentiation and histone H3K9 acetylation in myocardial tissues as evidenced by increased α‐SMA expression and elevated levels of Ac‐H3K9. Post‐treatment of hypertensive mice with either C646 or L002 reverses hypertension‐induced H3K9 acetylation and myofibroblast differentiation (Figure 2C). This observation is highly significant as firstly, it confirms that ATp300 inhibitors L002 and C646 are acting through epigenetic modifications; and secondly, differentiation of resident cardiac fibroblasts or endothelial cells to myofibroblasts and increased synthesis and secretion of collagen by myofibroblasts are major events in the progression of fibrogenesis [reviewed in 2, 12].

The reversal of myofibroblast differentiation or conversion of excessive collagen producing myofibroblasts to fibroblasts or collagenase producing senescent myofibroblasts^12^, ^13^ by ATp300 inhibitors is highly significant in the context of therapy. Additionally, we have previously shown that treatment of human cardiac fibroblasts with ATp300 inhibitor C646 blocks TGF‐β‐induced collagen synthesis.^11^ Interestingly, the levels of p53 are significantly elevated in C646‐treated cardiac fibroblasts (Figure S3). Previously, we have demonstrated that p53 is a negative regulator of profibrogenic signal‐induced Type I collagen synthesis [reviewed in 9] and a bona fide marker and mediator of cellular senescence.^14^ Therefore, decrease in collagen synthesis in human cardiac fibroblasts by ATp300 inhibitor may be due to dual effects of inhibition of ATp300, an essential positive epigenetic regulator of Type I collagen synthesis and activation of p53, a negative regulator of TGF‐β‐induced collagen synthesis. Additionally, we cannot rule out the conversion of collagen producing myofibroblasts to collagenase producing senescent myofibroblasts by ATp300 inhibitor‐induced p53, a most potent inducer of fibroblast senescence. In order to understand in depth the molecular mode of action of ATp300, it will be interesting to determine the role of ATp300 inhibitor in myofibroblast senescence and expression profile of matrix proteins and matrix protein degrading enzyme genes. Such studies are in progress in our laboratory. Our in vivo data on reversal of α‐SMA expression and acetylation of H3K9 by ATp300 inhibitors is in agreement with in vitro data showing induction of myofibroblast differentiation and elevated levels of Ac‐H3K9 in ATp300 overexpressed cardiac fibroblasts and its inhibition by ATp300 inhibitors C646 and L002 [unpublished data]. Additionally, C646 effectively reduces TGF‐β‐induced myofibroblast transition of vascular endothelial cells and renal epithelial cells in vitro during EndMT and EMT respectively [data not shown]. Both EndMT and EMT‐derived myofibroblast‐like cells are known to contribute significantly to fibrogenesis.^15^ The in vivo data presented in this study verify the effectiveness of ATp300 inhibitors in halting or reversing profibrogenic signal‐induced histone acetylation, myofibroblast differentiation and collagen synthesis, the major events during fibrogenesis.

In summary, this short communication presents highly significant data on the reversal of hypertension‐induced cardiac pathologies tested by treating hypertensive mice with ATp300 specific small molecule inhibitors; and establishes a solid foundation for epigenetic treatment of high blood pressure‐induced cardiac fibrosis using small molecule inhibitors targeting druggable ATp300. As both ATp300 inhibitors failed to reduce high blood pressure, ATp300 inhibitors will therapeutically be more effective in combination with blood pressure lowering drugs. Further studies are warranted to identify more efficient drug‐like small molecule inhibitors of ATp300 and their application in preclinical setting and for future clinical trials of hypertension‐induced cardiac pathology.

## CONFLICT OF INTEREST

There is no potential conflict of interest.
