# Supplementary figures and images for: Acetyltransferase p300 inhibitor reverses hypertension‐induced cardiac fibrosis
Source: J Cell Mol Med. 2019 Feb 1;23(4):3026–31. doi: 10.1111/jcmm.14162 (PMC6433695; doi:10.1111/jcmm.14162)

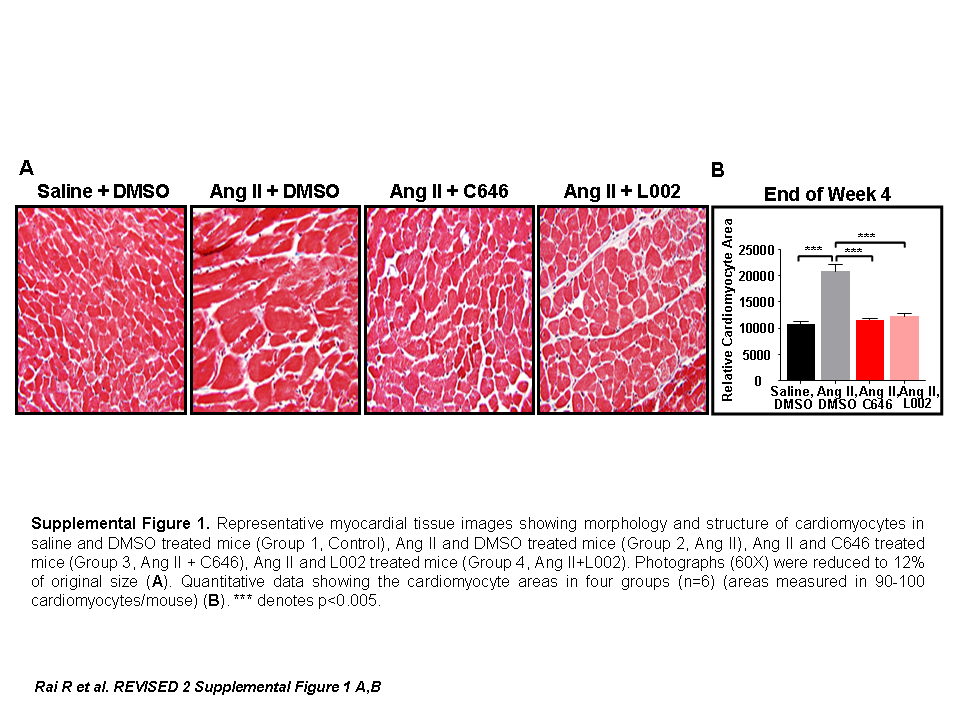

Supplement: Supplementary file 1 [file JCMM-23-3026-s001.tif]

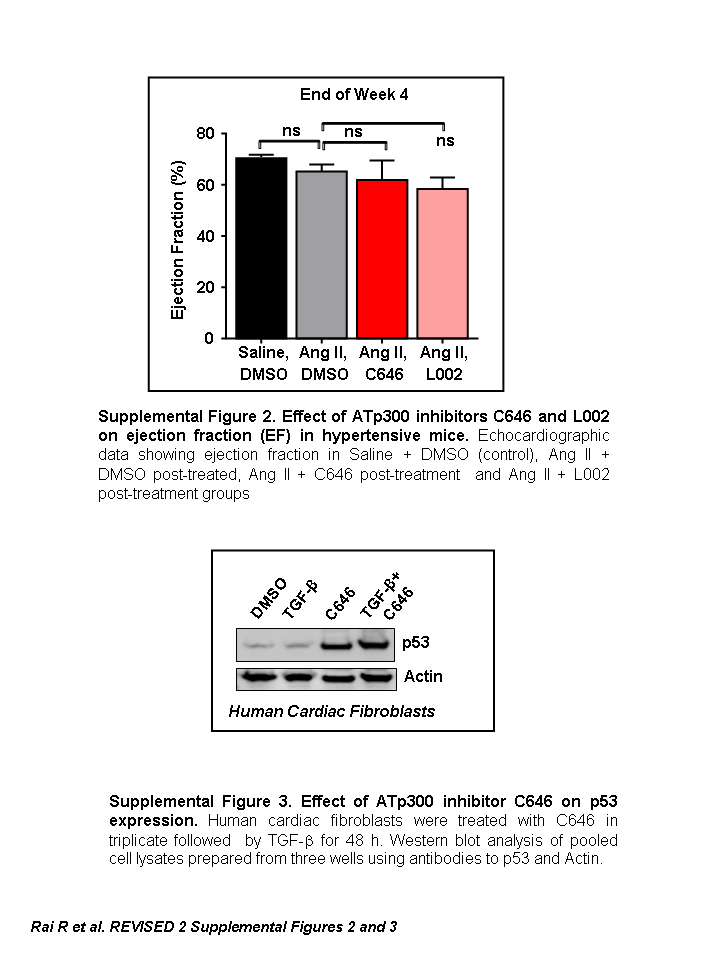

Supplement: Supplementary file 2 [file JCMM-23-3026-s002.tif]
